# Symmetrical peripheral gangrene following testicular torsion surgery: A case report

**DOI:** 10.1002/ccr3.8506

**Published:** 2024-02-08

**Authors:** Kamoga Dickson, Kato Ronald

**Affiliations:** ^1^ Department of Emergency Medicine Mulago National Referral Hospital Kampala Uganda; ^2^ Department of Emergency Medicine The Savannah Hospital Nairobi Kenya

**Keywords:** angiogram, disseminated intravascular coagulation, symmetrical peripheral gangrene, testicular torsion

## Abstract

**Key Clinical Message:**

Symmetrical peripheral gangrene (SPG) is very rare condition associated with symmetrical ischemia and gangrene affecting two or more distal extremities. It is almost always associated with septicemia and has a high mortality rate. The rarity of this condition and lack of prospective trials makes its recognition and management difficult. Care providers should maintain a high index of suspicion for SPG in patients with sepsis who develop cyanosis and ischemia of extremities. Doing early culture and sensitivity studies is key in guiding apropriate antibiotic treatment.

**Abstract:**

Symmetrical peripheral gangrene (SPG) is very rare condition associated with symmetrical ischemia and gangrene affecting two or more distal extremities. It can occur at any age and may affect either sex. It is almost always associated with septicemia and has a high mortality rate (up to 35%). The rarity of this condition and lack of prospective trials makes its recognition and management difficulty. Only a few case reports have been in literature since its discovery in 1981. A 14 year old boy was referred to our tertiary facility due to postoperative wound sepsis. He had undergone right scrotal exploration and orchidectomy due to right testicular torsion. His initial symptoms were abrupt onset of scrotal pain and swelling which he developed while playing. Physical examination findings on admission were severe pallor of mucous membranes, fever and diaphoresis and mild respiratory distress. He also had a gangrenous perineal area involving the penis and cyanosed tips of fingers and toes bilaterally. He had a normal ankle branchial index of 0.9. His preliminary laboratory investigations revealed a marked neutrophilia, anemia, thrombocytopenia, and elevated D‐Dimers. Initial resuscitative interventions included oxygen therapy, blood transfusion with whole blood and platelets, empirical antibiotics, analgesics, and surgical debridement of the perineal in theater. A diagnosis of severe sepsis complicated with disseminated intravascular coagulation (DIC) was made. The cyanosis on extremities spread proximally during the patient's course of treatment to full blown gangrene. At the time when clinical and biochemical remission of the infection was attained, the gangrene had demarcated at below elbow in both upper limbs and below knees in both lower limbs. An arterial angiogram was done and revealed normal flow in all proximal and distal branches of the aorta with no occlusion. A multidisciplinary agreement to conduct quadrilateral amputations plus penile amputation was made between urologists, vascular and orthopedic surgeons. The exact pathogenesis of how SPG occurs is not well understood. The underlying mechanism includes a low flow state with DIC. Ischemic changes usually begin in the peripheries and extend proximally. Ischemic changes are not preceded by peripheral vascular occlusive disease. SPG should be suspected when a patient present with marked coldness, pain in the distal extremities, cyanosis, and pallor. Early recognition helps to arrest the progression of ischemic changes before overt gangrene occur and improves the qaulity of life.

## 1 INTRODUCTION

Symmetrical peripheral gangrene (SPG) was initially described in 1981 by Hutchinson.^1^ SPG commonly occurs following bacterial infections like *Staphylococcal, Pneumococcal, Meningococcal, Streptococcal, E. coli*, Pseudomonal septicemia, Viruses or Rickettsial infection. The current case under consideration involves an exceptionally uncommon manifestation of the already rare condition. This condition arose as a result of testicular torsion, which subsequently resulted in Fournier's gangrene. The gangrene, in turn, gave rise to systemic sepsis, leading to disseminated intravascular coagulation (DIC), characterized by the deposition of microvascular thrombi in the distal extremity microcirculation, ultimately resulting in ischemia and gangrene.

Less commonly it follows after usage of drugs like ergot, vasopressin, noradrenaline, thiopentone or inadvertent intramuscular injection gone intra‐arterially. Conditions like Polymyalgia rheumatica, Sickle cell disease, Raynaud's phenomenon, diabetes mellitus, cryoglobulinemia and inherited coagulopathies like Protein‐C, S and Antithrombin‐3 deficiency can also cause SPG.

The exact pathogenesis of how SPG occurs is not well understood. The underlying mechanism includes a low‐flow state with DIC.^1^ DIC results in the formation of widespread systemic thrombi that subsequently get deposited in the microcirculation of the extremities. Effective treatment hinges on promptly recognizing and addressing the underlying cause to prevent the progression of ischemia into established gangrene.

The use of anticoagulants and thrombolytics is a topic of debate in managing SPG due to the suspected involvement of DIC, which depletes platelets. Administering these agents may carry the risk of severe bleeding and should be evaluated on a case‐by‐case basis, taking into consideration the patient's bleeding risk. It is strongly recommended to conduct trials of plasmapheresis and leukapheresis to ascertain their specific roles in managing SPG and establish treatment objectives.

Our patient sought treatment while the gangrene was still in the evolution phase. We initiated treatment for sepsis with broad spectrum antibiotics early, but the gangrene continued to spread possibly. This was partly due to our limited understanding of this complication and the absence of conclusive evidence regarding effective treatments.

## 2 CASE REPORT

### 2.1 History and clinical presentation

We present a case of 14 year‐old boy who was referred to our tertiary facility from a mission hospital in a refugee camp. Prior to admission at our facility, he had undergone right scrotal exploration and orchidectomy due to right testicular torsion. He was referred due to wound sepsis postoperatively. His initial symptoms were abrupt onset of scrotal pain and swelling which he developed while playing. He was first treated from home with local remedies for close to 24 h but without improvement which prompted his parents to take him to the mission hospital where the first surgery was done.

On arrival to our ED; he was in mild respiratory distress, with tachypnea, he was tachycardic at 124 bpm and had pallor of mucous membranes. His BP was normal at 109/68 mmHg, his neck veins were not distended, and he had normal heart sounds. He was diaphoretic and hyperrexic at 38.70°C. The tips of his fingers and toes appeared cyanosed bilaterally and had strikingly low peripheral oxygen saturation on pulse oximetry (widely fluctuating between 30% and 20% on room air) which wasn't in keeping with the clinical picture. He had a normal random blood sugar of 9 mmL/L. There were ecchymoses under the skin overlying the extremities and he bleed easily from puncture sites. The right groin area appeared necrotic and septic. He had no known chronic illnesses, wasn't on any chronic medications and he died being a smoker.

## 3 METHODS

### 3.1 Differential diagnosis

1. Sepsis complicated with DIC and anemia.
2. Raynaud's phenomena because of the peripheral cyanosis; however, it was the index onset of symptoms and was not related to change in the weather.
3. Thomboangitis obliterans.
4. Vasculitic gangrene.
5. Thromboembolic gangrene.
6. Calciphylaxis.

### 3.2 Investigations and treatment

We initiated our resuscitative interventions with oxygen supplementation by a non‐rebreather face mask at 15 L/min which improved his SPO2 up to 99% but would still occasionally drop to less than 80% despite the patient not being visibly dyspneic which raised concerns of peripheral hypoperfusion. We inserted a large bore cannula and started intravenous fluid resuscitation with crystalloids, took off a blood sample for preliminary laboratory investigations. The following results were obtained;

WBC; 16 × 10^3^/μL (4–12), Neutrophils; 10.8 × 10^3^/UL (1.7–7.7), Hb; 4.8 g/dL (11–17 g/dL), PLT 26 × 10^3^/UL (150–450), MCV; 91.4 fL (71.0–97.0), MCH; 28.8 Pg (23.0–34.0), liver function tests—normal, blood slide for malaria—negative, HB electrophoresis—normal, serum electrolytes—normal, renal function tests—normal.

The aforementioned tests revealed significant increases in neutrophil count, as well as severe reductions in platelet count and red blood cell levels.

Based on these findings, a diagnosis of severe sepsis complicated by DIC and anemia was made, with the infection focus being the surgical site for the testicular torsion.

To address this, the patient was initiated on broad‐spectrum empirical antibiotics (intravenous amoxiclav and metronidazole). Additionally, the patient received a transfusion of 3 units of whole blood at a dose of 20 mL/kg and platelets at 15 mL/kg. Intravenous paracetamol was administered to manage fever at a dosage of 15 mg/kg. The patient was scheduled for surgical debridement of the perineal area in the operating theater.

Following the surgery, the patient was admitted to the high dependency unit and continued to receive supplemental oxygen, the same antibiotics, and IV paracetamol. However, in the postoperative period, cyanosis in the fingers and toes became more pronounced. Over the subsequent days, it progressed into fully developed gangrene, seemingly spreading towards the body's core. The patient continued to exhibit a high heart rate of 132 beats per minute, persistent fever at 37.8°C despite antipyretics and antibiotics, increased respiratory rate, profuse sweating, and developed signs of delirium. Due to the escalating oxygen requirements and increasing work of breathing, the decision was made to intubate the patient and initiate mechanical ventilation. Subsequently, the patient was transferred to the intensive care unit and placed on mechanical ventilation on assist control mode.

Additional laboratory tests were done and the following results were obtained; and listed as follows; WBC–15.95 × 10^3^/μL (3.20–9.00), HB–6.4d/dl (9.8–17.0), PLT–85 × 10^3^/μL (150–450), Neut–6.7893 × 10/μL (1.7–7.7). Blood Culture and Sensitivity—Negative, APTT—25.9 s (24.0–38.0), APTTR—0.82 (0.77–1.23), D‐Dimers 850 ng/mL (<500), serum electrolytes, LFRTs, RFTs—all normal.

We made a change in his antibiotic treatment to intravenous meropenem at a dose of 500 mg every 8 h. Additionally, we administered another unit of whole blood. Our continued approach included maintaining him on mechanical ventilation, providing antipyretics, administering a dextrose bolus, periodic nasogastric tube feeds, and ensuring he received maintenance IV fluids. The patient's clinical condition showed significant improvement, leading to his extubation on the 6th day, with his neurological and cardiorespiratory functions returning to normal.

**FIGURE 1:**
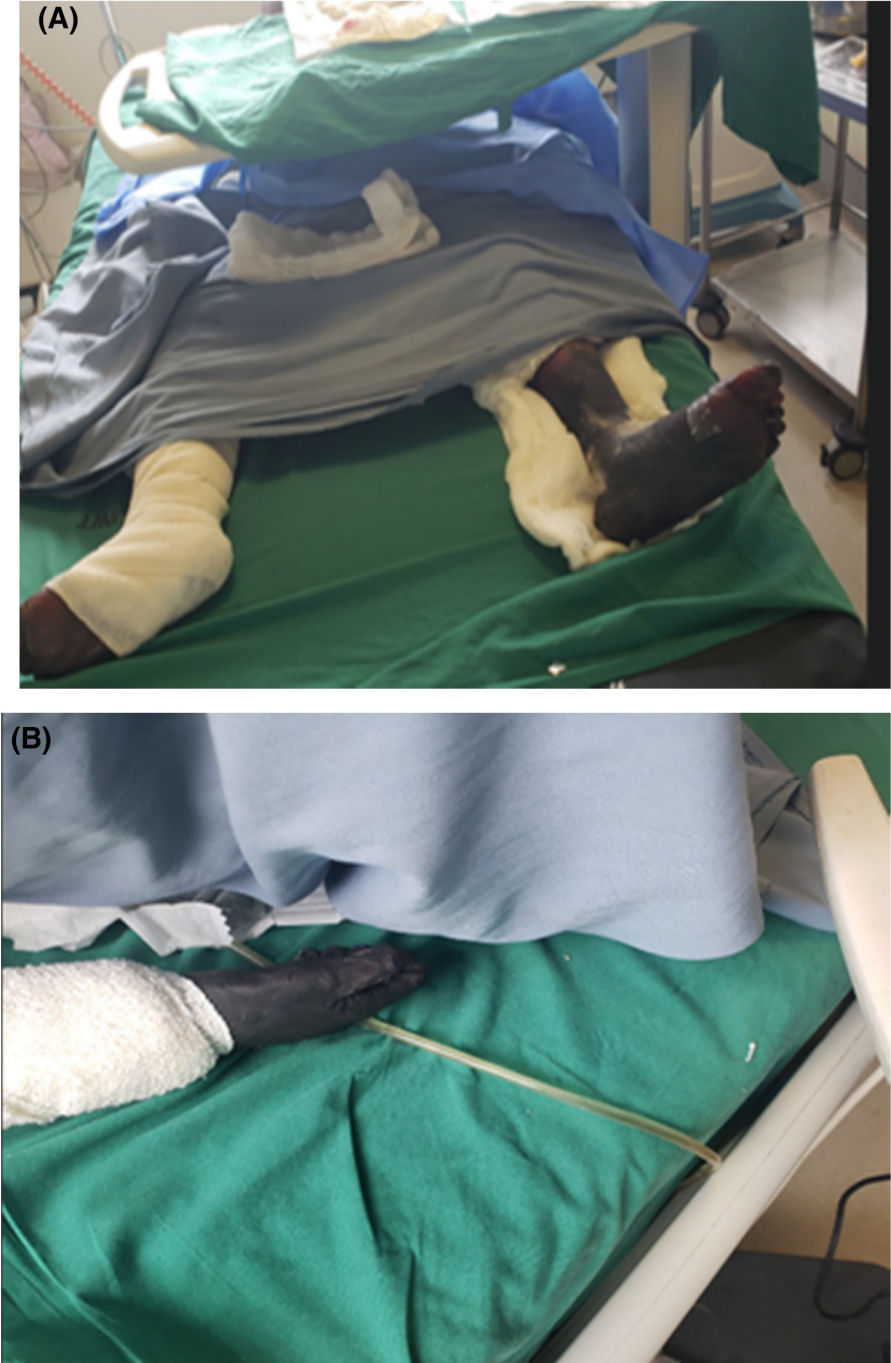
(A) Showing gangrene of both lower limbs that had formed clear boundaries below the knees. This was gross appearance of the limbs pre‐operatively. Patient was clinically stable without fever. (B) Showing upper limb gangrene of the hand. It was well‐demarcated below the elbow. This was after the patient's clinical status had stabilized and had been scheduled for amputation.

The gangrene had clearly defined boundaries, symmetrically located below the knees and below the elbows in all extremities (Figure 1A,B). The perineal (Fournier's) gangrene also exhibited well‐defined demarcation. Over the following week, the patient's vital signs consistently remained within the normal range, and his white blood cell count returned to normal levels. We discontinued the use of antibiotics and sent out an orthopedics consultation to explore the possibility of quadrilateral amputations.

**FIGURE 2:**
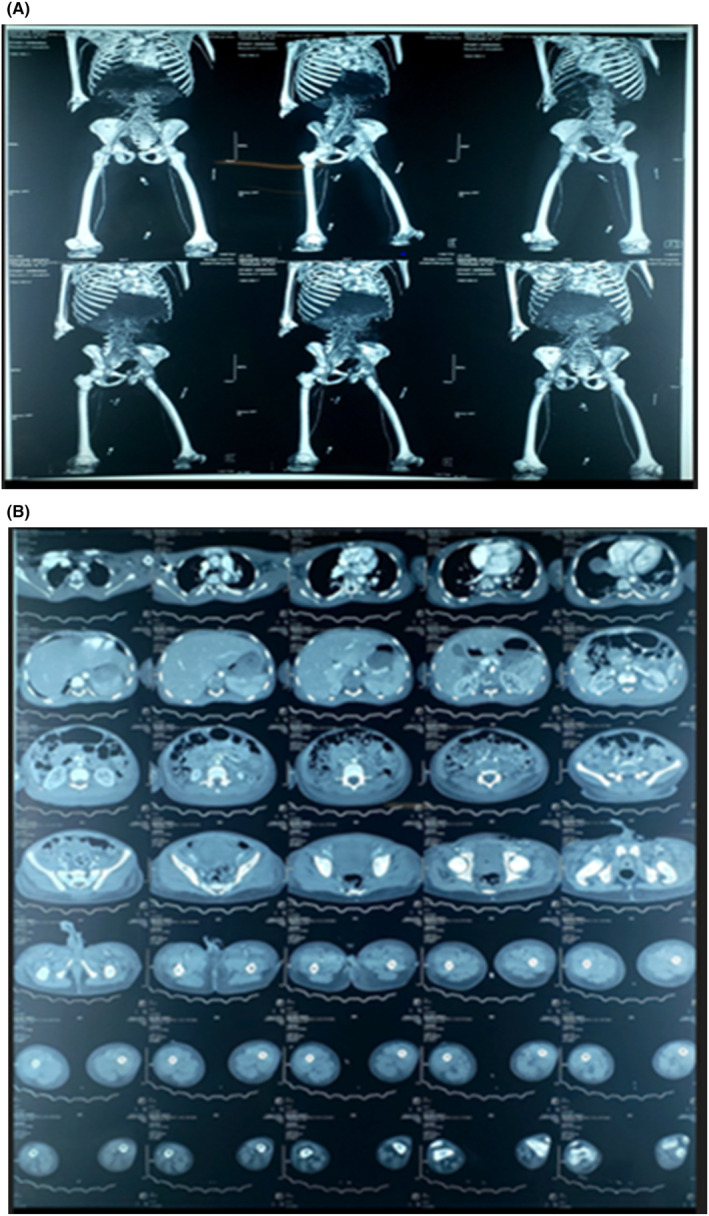
(A) 3D reformats of the aorta and its distributions showing on obstruction. (B) An enhanced helical chest and abdominal CT scan using an angiogram protocol demonstrated well‐sustained contrast enhancement throughout the thoracic and abdominal aorta, with no evidence of linear filling defects, mural thrombi, or atherosclerotic plaques. The aorta exhibited normal diameters at all assessed levels.

An aortic angiogram was done which revealed no occlusion in either the lower and upper limbs major arteries (Figure 2A). 3D reformats of the aorta and its distributions showing on obstruction was demonstrated (Figure 2B). Under multidisciplinary approach involving the orthopedic team, vascular surgeons, and urologists the penis, the lower limbs at the level of mid‐thighs and upper limbs above elbow based on the level of demarcation of the gangrenous area were amputated.

### 3.3 Conclusion

This case involves a 14‐year‐old male admitted for sepsis following testicular torsion surgery. The sepsis was complicated by DIC and anemia. The patient initially presented with fever, respiratory distress, and bilateral cyanosis of figures and toes. Baseline tests revealed leukocytosis, anemia, and significant thrombocytopenia. The cyanosis spread proximally and developed into full‐blown symmetrical gangrene. After 10 days of aggressive antibiotic treatment, the gangrene's advancement was successfully halted, and demarcation occurred below the elbows in the upper limbs and below the knees in the lower limbs. Consequently, amputation was performed on all affected limbs.

## 4 CASE DISCUSSION

SPG is a relatively rare condition characterized by symmetrical ischemia and gangrene occurring in the distal extremities. It can manifest at any age and is not limited to a specific gender. Managing SPG requires a proactive approach due to its significant association with elevated morbidity and mortality rates. The majority of patients ultimately undergo amputation of the affected limbs. Maintaining a high level of suspicion and promptly identifying the underlying cause are crucial in order to preserve both life and limb before irreversible ischemia and gangrene develop. SPG typically involves two or more extremities and is not linked to the obstruction or inflammation of large blood vessels.^1^

SPG was initially described in 1981 by Hutchinson. SPG commonly occurs with bacterial infections like *Staphylococcal, Pneumococcal, Meningococcal, Streptococcal, E. coli*, Pseudomonas septicemia, Viruses or Rickettsial infection.^2^ The case under discussion aligns with the typical occurrence of this rare phenomenon Symmetrical Peripheral Gangrene (SPG) as it manifested as a complication of sepsis which followed testicular torsion surgery. Less commonly it follows after usage of drugs like ergot, vasopressin, noradrenaline, thiopentone or inadvertent intramuscular injection gone intra‐arterially. Conditions like Polymyalgia rheumatica, Sickle cell disease, Raynaud's phenomenon, diabetes mellitus, cryoglobulinemia, and inherited coagulopathies like protein‐C, S and Antithrombin‐3 deficiency can also cause SPG.^3^

The exact pathogenesis of how SPG occurs is not well understood. The underlying mechanism includes a low flow state with DIC. Molos et al noted DIC as an important underlying factor in 85% of patients who develop SPG. Davis et al reported similar findings in his cohort of 12 patients with SPG. SPG manifests unfavorably in patients with immunosuppression, asplenism, hypothermia, diabetes mellitus, and renal failure.^4^ The case being reported didn't have immunosuppression. SPG should be suspected when a patient presents with marked coldness, pain in the distal extremities, cyanosis, and pallor. Early recognition helps to arrest progression of ischemic changes before overt gangrene occurs. Ischemic changes usually begin in the peripheries and extend proximally. Ischemic changes are not preceded by peripheral vascular occlusive disease. The distal pulses in the large vessels are intact.^2^ DIC and an associated low flow state may result in occlusion of the microcirculation in the affected limbs with resultant ischemia and gangrene.

SPG may have a mortality rate as high as 35% with most patients inevitably requiring an amputation.^4^ Initially, a nonsurgical approach is appropriate with emphasis on stabilizing the patient and treating the underlying condition. This allows the gangrene to demarcate before an amputation is done.

Pathological evaluation of amputated specimens has shown thrombi which are mainly found in small vessels sparing the large vessels. There is no associated vasculitis as well.^5^ This is important as focus should not be on the distal ischemic changes only without addressing the underlying disease process.

Despite a wide array of etiological causes for SPG, it is not uncommon to fail to identify an underlying cause. This may be particularly challenging in a low resource setting where discriminatory investigations to identify the cause may be difficult to undertake.^6^ Kudzai et al highlighted in a case report that lack of diagnostic investigations should not compromise urgent and timely management of SPG and that delay in initiating management for patients with SPG may result in poor outcomes including loss of limbs and death.

Our case in study faced great limitations in the early recognition and management because of majorly lack of high suspicion index for its occurrence. At presentation, the patient already had overt signs of SPG manifesting with the peripheral cyanosis and ischemia of finger tips and toes. He had intact peripheral pulses. Early recognition and targeted treatment initiation would likely change the patient's prognosis. Management efforts were initially directed towards establishing a vascular occlusive disease as a cause of the patient's gangrene which drastically shifted to watchful waiting until the gangrene became clearly demarcated and quadrilateral amputations were done. The initial antibiotics that the patient received weren't specifically targeting the causative organism and blood culture results were obtained late after the gangrene had spread.

Some of the common differentials for SPG including thomboangitis obliterans, thromboembolic gangrene, calciphylaxis, and vasculitic gangrene^3^ were unlikely causes in our patient. These were ruled out through history and investigations. It was the index onset of symptoms making a diagnosis of Raynaud's phenomenon unlikely and the patient tolerated the cold very well. Patient had never smoked and denied eating moldy grain or other food staffs making thromboarteritis obliterans and egort poisoning respectively unlikely.

The choice of investigations for patients with suspected SPG should be guided by clinical features and must be individualized to each patient. An infection screen is important to rule out sepsis and DIC. Blood investigations like a full blood count, a blood culture, and a peripheral smear are useful to assess for sepsis. Our patient had a well‐established systemic infection and DIC evidenced by a neutrophilic leukocytosis, elevated D‐Dimers and thrombocytopenia on his preliminary investigations. A blood culture was negative possibly because it was done days after broad‐spectrum antibiotic initiation. A blood smear for malaria parasites was negative which ruled out malaria as a cause for this patient's SBP. An arteriogram highlighted absence of involvement of large peripheral vessels.

To this end, management recommendations for SPG involves use of broad spectrum antibiotic cover particularly in patients with sepsis and DIC.^7^ Patients who develop a bleeding disorder should have it corrected with replacement of depleted clotting factors.^1^ Prostacyclin (epoprostenol) and tissue plasminogen infusion have been used with some success in managing patients with SPG. Plasmapheresis, leukapheresis, and sympathetic blockade may be beneficial but have a limited role in SPG.^5^ Use of anticoagulants like heparin and aspirin is controversial. Johansen and Hansen reported that heparin, aspirin, and streptokinase were not effective in preventing progression of gangrene when used in patients with SPG. The addition of oral corticosteroids did not confer any treatment benefit as well.^6^

In this case study, bacterial colonization ensued after testicular torsion surgery under seemingly not ideal aseptic techniques at a mission Hospital. Local infection led to Fournier's gangrene. The bacteria spread hematogenously leading to systemic sepsis. The widespread systemic inflammation led to intravascular fibrin activation and tissue factor mediated thrombin generation. Activation of intravascular fibrin and tissue factor leads to microvascular thrombi deposition causing ischemia and gangrene.^7^

## 5 CONCLUSION

SPG is a rare condition with no universally proven treatment. Management varies for each patient, focusing on underlying causes. Controversy surrounds the use of anticoagulants and thrombolytics due to potential bleeding risks tied to DIC. Clinical trials exploring the role of Plasmapheresis and leukapheresis in management are recommended. Clinicians should maintain a high suspicion for SPG, especially in septic patients with unexplained peripheral cyanosis and ischemia.

## AUTHOR CONTRIBUTIONS

**Kamoga Dickson:** Conceptualization; investigation; methodology; visualization; writing – original draft; writing – review and editing. **Kato Ronald:** Formal analysis; methodology; project administration; supervision; visualization.

## FUNDING INFORMATION

There was no funding or any financial assistance that was contributed towards this manuscript.

## CONFLICT OF INTEREST STATEMENT

There is no any conflict of interest between authors and all our institutions in regarding to publication of this report.

## CONSENT

A written informed consent was obtained from the patient to publish this report in accordance with the journal's patient consent policy. From the patient, “I confirm that, the case report has been fully explained to me and all my questions have been answered to my satisfaction, I therefore have no objection to the publication of this report.”

## Data Availability

The data that support the findings of this study are openly available in PubMed (https://pubmed.ncbi.nlm.nih.gov).
